# Tetralogy of Fallot Surgical Repair: Shunt Configurations, Ductus Arteriosus and the Circle of Willis

**DOI:** 10.1007/s13239-017-0302-5

**Published:** 2017-04-05

**Authors:** Senol Piskin, Gozde Unal, Ahmet Arnaz, Tayyar Sarioglu, Kerem Pekkan

**Affiliations:** 1 Department of Mechanical Engineering, Koç University, Rumeli Feneri Kampüsü, Sarıyer, Istanbul, Turkey; 2 Faculty of Engineering and Natural Sciences, Sabancı University, Tuzla, Istanbul, Turkey; 3 Department of Cardiovascular Surgery, Acıbadem Bakırköy Hospital, Istanbul, Turkey; 4 Department of Pediatric Cardiovascular Surgery, School of Medicine, Acıbadem University, Istanbul, Turkey

**Keywords:** Congenital heart disease, Blalock Taussig shunt, Circle of Willis, Pre-surgical planning, Hemodynamics, Computational fluid dynamics

## Abstract

In this study, hemodynamic performance of three novel shunt configurations that are considered for the surgical repair of tetralogy of Fallot (TOF) disease are investigated in detail. Clinical experience suggests that the shunt location, connecting angle, and its diameter can influence the post-operative physiology and the neurodevelopment of the neonatal patient. An experimentally validated second order computational fluid dynamics (CFD) solver and a parametric neonatal diseased great artery model that incorporates the ductus arteriosus (DA) and the full patient-specific circle of Willis (CoW) are employed. Standard truncated resistance CFD boundary conditions are compared with the full cerebral arterial system, which resulted 21, −13, and 37% difference in flow rate at the brachiocephalic, left carotid, and subclavian arteries, respectively. Flow splits at the aortic arch and cerebral arteries are calculated and found to change with shunt configuration significantly for TOF disease. The central direct shunt (direct shunt) has pulmonary flow 5% higher than central oblique shunt (oblique shunt) and 23% higher than modified Blalock Taussig shunt (RPA shunt) while the DA is closed. Maximum wall shear stress (WSS) in the direct shunt configuration is 9 and 60% higher than that of the oblique and RPA shunts, respectively. Patent DA, significantly eliminated the pulmonary flow control function of the shunt repair. These results suggests that, due to the higher flow rates at the pulmonary arteries, the direct shunt, rather than the central oblique, or right pulmonary artery shunts could be preferred by the surgeon. This extended model introduced new hemodynamic performance indices for the cerebral circulation that can correlate with the post-operative neurodevelopment quality of the patient.

**Electronic supplementary material:**

The online version of this article (doi:10.1007/s13239-017-0302-5) contains supplementary material, which is available to authorized users.

## Introduction

The primary surgical repair of common congenital heart defects (CHD), particularly the tetralogy of Fallot (TOF), pulmonary artery atresia (PAA), and hypoplastic left heart syndrome (HLHS), involve the reconstruction of palliative vascular shunts that are anastomosed between the aorta and the pulmonary arteries (1st stage shunt surgery). Studies demonstrated poor functional outcome with reduced exercise capacity, diminished cardiac output, and risks of heart failure after the surgical repair.^10^,^53^,^54^ Furthermore, the post-operative shunt hemodynamics is not stable due to vascular growth, collateral vessels, and the post-operative management strategy.^13^,^38^

Although there is no consensus on the hemodynamic evaluation criteria that can correlate with the post-operative performance of the 1st stage shunt surgeries,^50^ computational fluid dynamics (CFD) simulations are proven useful in improving the hemodynamics of the 3rd stage shunt surgeries.^9^ Similar studies that investigate the hemodynamics of the 2nd stage shunt surgeries^7^,^40^ as well as the 1st stage surgical palliation are relatively rare.^3^–^5^,^23^,^24^,^30^,^44^ Furthermore the existing studies focused entirely on the surgical repair of HLHS^48^ in which the Norwood (innominate artery or the aorta shunt to the right pulmonary artery) and Sano (right ventricle to pulmonary artery shunt) shunt variations are analyzed.^30^ However the 3D arterial geometry of CHD constitutes a spectrum of anatomical templates,^39^ in which HLHS represents only one of the extreme anatomical configurations. The other opposite anatomical extreme is the TOF disease anatomy.^1^,^19^ Unlike HLHS, the TOF disease template has a large aorta and under developed pulmonary arteries. It is composed of a ventricular septal defect, right ventricular hypertrophy^2^,^16^ and occurs in 3 out of 10,000 live births.^2^ Thus, the lack of comprehensive 1st stage shunt surgery planning investigations on TOF disease anatomy motivates the present manuscript.

Another focus of the current study is the Circle of Willis (CoW) region of the anatomy since the computational studies available from literature that investigate the first-stage shunt surgeries overlooked the full detail of the cerebral great vessel circulation system, particularly the CoW section,^32^ to our knowledge. In this manuscript we hypothesize that the neonatal cerebral arterial hemodynamics is critical for the 1st stage patient-specific pre-surgical shunt design. Unlike the adult cerebral circulation, for the newborn baby a large percent of the total cardiac output may be delivered to the brain—50% in neonates versus 15% in adults.^41^ Optimal intra- and post-operative cerebral arterial perfusion is also critical for the normal neurodevelopment of the newborn CHD patient. Among fetuses with single ventricle anomalies, lower cerebrovascular resistance was associated with higher neurodevelopmental (ND) scores.^55^ Indeed, ND dysfunction has become the most common and potentially the most disabling outcome of CHD repair,^34^ including high prevalence of low-severity developmental problems in the areas of language, motor skills, attention, and executive function.^33^ Neurodevelopment-associated impairment may occur in up to 70% of survivors as they grow through childhood.^26^ These recent clinical facts^18^ prompted the present investigation where potential cerebral perfusion changes due to different shunt configurations are studied in detail.

Patent ductus arteriosus (PDA) accounts for approximately 10% of all CHDs with an incidence of at least 2–4 per 1000 term births.^14^ DA plays an important role in the 1st stage palliation of congenital heart diseases and can be left open clinically both pre and post- operative stages as a hybrid therapy.^14^ Although a moderate size patent DA should be closed by the time the patient is 1–2 years old, the decision of DA closure at the neonatal period remains uncertain.^6^ In the present manuscript, we studied both the patent DA and closed DA states in order to provide insight to the surgeons on the role of DA in shunt hemodynamics.

Thus, in this manuscript we study the pre-surgical planning of 1st stage shunt operations through a 3D model of cardiovascular system including neck and cerebral arteries (CoW region). Resistance boundary conditions are assigned *both* to the artery outlets and inlets to represent the flow competition between the outflow trunks and the downstream organs. Three different shunt configurations designed by pediatric cardiovascular surgeons are implemented into our model. Furthermore, these shunt configurations are studied in two different PDA stats: when the DA is open and functioning and when the DA is totally closed and not functioning.

The present manuscript is organized as follows; in “Materials and Methods” section, together with the details of the CFD solver, the 3D reconstruction of realistic geometry cardiovascular system and CoW region and shunt configurations designed by surgeon as post-surgery anatomy post-surgery anatomy for surgical planning are described. In “Results” section, flow splits and wall shear stress (WSS) distributions are presented for the three shunt configurations using TOF disease template including CoW region. Also the closed and patent DA states are examined for all three shunt configurations. In “Discussion” section, the post-surgery results of shunt configurations and DA states are compared and analyzed in detail. The limitations, assumptions of our approach and their adequacy are provided at the separate “Limitations” section. Finally, the surgical interpretations of the key findings are stated in “Conclusions” section.

## Materials and Methods

### 3D Geometry (Aortic Arch, Neck and Cerebral Arteries)

**Figure 1:**
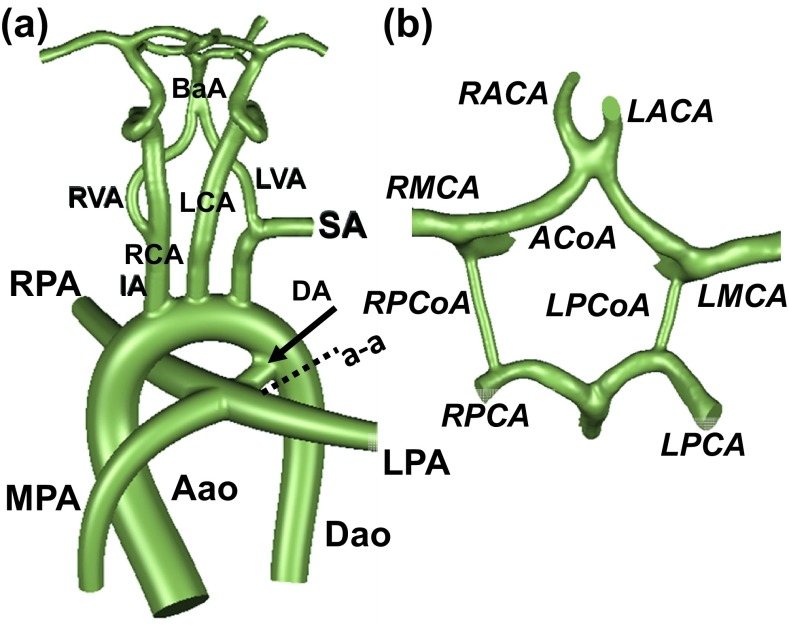
(a) Realistic anatomical model of neonatal great arteries of tetralogy of Fallot (TOF) coupled to the Circle of Willis (CoW), close-up shown in (b). Section a–a, shown in dashed lines indicates aortic isthmus; the velocity profile location of mesh convergence figure (see Supplementary Material, Figure B). (Aao: Ascending aorta, Dao: Descending aorta, MPA: Main pulmonary artery, RPA: Right pulmonary artery, LPA: Left pulmonary artery, IA: Innominate artery, SA: Subclavian artery, RVA: Right vertebral artery, LVA: Left vertebral artery, RACA: Right anterior cerebral artery, LACA: Left anterior cerebral artery, RMCA: Right middle cerebral artery, LMCA: Left middle cerebral artery, RPCA: Right posterior cerebral artery, LPCA: Left posterior cerebral artery.) The CoW region features the anterior communicating artery (ACoA), the right posterior communicating artery (RPCoA) and left posterior communicating artery (LPCoA). The cerebral arteries have six outlets: RACA, LACA, RPCA, LPCA, RMCA and LMCA. The coupling of both anatomical models is performed using our in-house SketchCAD software.^12^ The reconstructed neck arteries include the right carotid artery (RCA), left carotid artery (LCA), RVA, LVA and basilar artery (BaA). This complex arterial manifold has two inlets; Aao and MPA and five outlets; Dao, RPA, LPA and three neck arteries (brachiocephalic artery (BA) or IA, LCA and SA). For simulations, the ductus arteriosus (DA) is included and connected to the transverse arch for simulating patent ductus (PDA) and removed in models that employ ligated DA.

A realistic 3D aortic arch anatomy of TOF is established based on our previous anatomical reconstructions^8^ where the left ventricle aortic outflow diameter is significantly less than normal, representing a symmetric diffuse stenosis^39^ (Fig. 1). Vessel dimensions of this idealized model have been validated rigorously and employed in our earlier hemodynamic investigations involving the neonatal stage.^8^,^27^,^28^,^37^ This anatomical template has a long publication history and its evolution is available in the Supplementary Material (Appendix A). For the present study, the anatomical TOF template was further improved by adding the cerebral arteries including the CoW using a magnetic resonance imaging (MRI) scan of a healthy young adult through approved institutional review board (IRB). The original cerebral geometry is scaled down 1.7 times, so that the connecting head-neck arteries are consistent with the neonatal aortic arch dimensions (Fig. 1). The cerebral anatomical dimensions are further validated here through the clinical neonatal cerebral measurements as summarized in the Supplementary Material (Appendix A). Nomenclatures of all the vessels involved are presented in Table 1. The coupled anatomy was created using Geomagic (Geomagic Inc., NC, USA).

**Table 1:** List of acronyms corresponding to the full names of the major vessels investigated.

Abbreviation	Full name	BC type
Arteries at aortic arch		
*Aao*	Ascending aorta	Resistance
*Ao*	Aorta	Inlet-Resistance
*DA*	Ductus arteriosus	Internal
*PDA*	Patent ductus arteriosus	Internal
*Dao*	Descending aorta	Resistance
*LPA*	Left pulmonary artery	Resistance
*RPA*	Right pulmonary artery	Resistance
*MPA*	Main pulmonary artery	Inlet-Resistance
Arteries belong to the neck region		
*IA*	Brachiocephalic (innominate) artery	Internal
*BaA*	Basilar artery	Internal
*SA*	(Left) subclavian artery	Resistance
*LCA*	Left carotid artery	Internal
*RCA*	Right carotid artery	Internal
*LVA*	Left vertebral artery	Internal
*RVA*	Right vertebral artery	Internal
Arteries belong to the cerebral region		
*ACoA*	Anterior communicating artery	Internal
*RPCoA*	Right posterior communicating artery	Internal
*LPCoA*	Left posterior communicating artery	Internal
*RACA*	Right anterior cerebral artery	Resistance
*LACA*	Left anterior cerebral artery	Resistance
*RMCA*	Right middle cerebral artery	Resistance
*LMCA*	Left middle cerebral artery	Resistance
*RPCA*	Right posterior cerebral artery	Resistance
*LPCA*	Left posterior cerebral artery	Resistance

Arteries are listed in three groups: great arteries proximal to the aortic arch, arteries associated with the neck region and cerebral arteries. The boundary condition (BC) types specified are also provided

### Shunt Configurations and Simulated Cases

**Figure 2:**
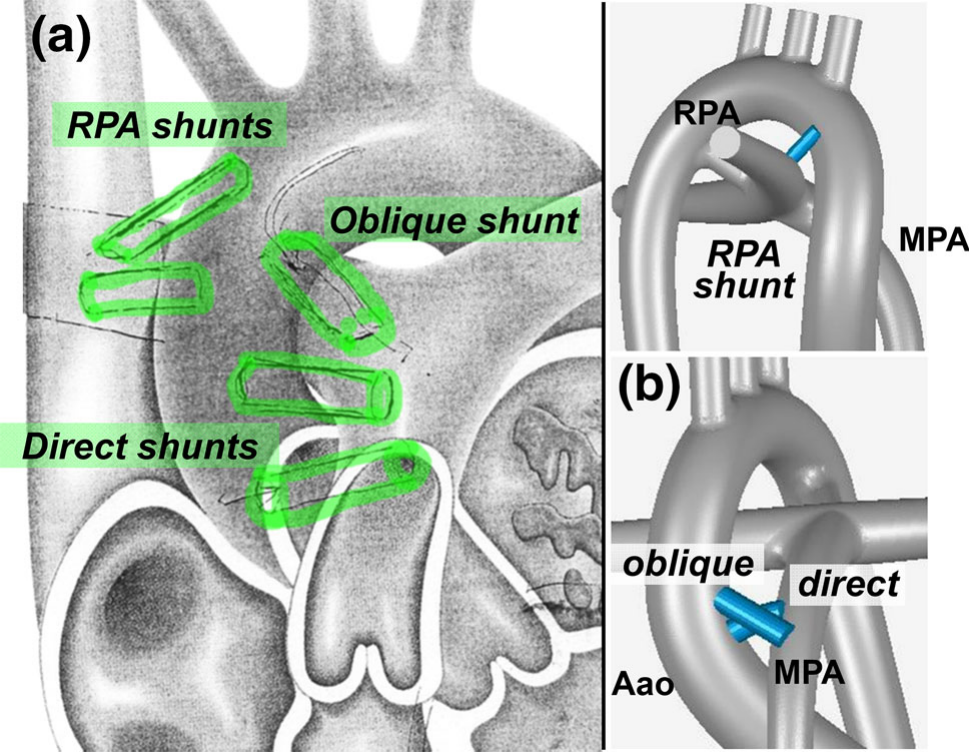
Shunt configurations analyzed in this study are illustrated on the tetralogy of Fallot (TOF) disease configuration; (a) Surgeon sketches of possible shunt configurations employed during the 1st stage surgical reconstruction. (b) The direct or “central” shunt configuration and its oblique version connecting ascending aorta (Aao) with the main pulmonary artery (MPA). The right pulmonary artery (RPA) shunt, shown on top, is configured proximal to the aortic arch and anastomosed to the RPA. RPA shunt resembles the traditional right-sided Blalock-Taussig configuration. Shunt lengths in the computational model are kept the same (5 mm) for unbiased performance comparison. DA vessel is patent in these 3D reconstructions.

The surgical shunts were created by 3D sketching on the computer using an in-house anatomical design toolkit.^12^ Several candidate shunt configurations were produced in collaboration with the two independent pediatric cardiovascular surgeons and 3 of those configurations were retained for the present study (Fig. 2). Two of these surgical configurations are central shunts constructed between the Aao and MPA. Central direct shunt (direct shunt) corresponds to a more horizontally configured case as opposed to a central oblique shunt (oblique shunt) case. The third configuration is a modified Blalock Taussig (mBT) shunt (RPA shunt) that connects the aortic arch and RPA that is retained as a baseline. The inner diameter of the implemented grafts is 2.5 mm polytetrafluoroethylene (PTFE) conduit.

All anatomical configurations and associated CFD simulation cases are summarized in Table 2. They include DA closure and TOF disease cases.

**Table 2:** Table of cases simulated for the present study.

Ductus arteriosus status	Configuration	Cases	
		w CoW	w/o CoW
Patent	Direct shunt	11	12
	Oblique shunt	21	22
	RPA shunt	31	32
Fully closed	Direct shunt	41	42
	Oblique shunt	51	52
	RPA shunt	61	62

6 cases are with Circle of Willis (w CoW) and 6 cases without Circle of Willis (w/o CoW) for tetralogy of Fallot (ToF) disease template

Inclusion of the full cerebral system improved the predictive capability of our simulations compared to the prior isolated aortic arch models in which the CoW flow characteristics were missing. These additional boundary condition verification simulations and the resulting performance improvements are summarized in the Supplementary Material (Appendix B) for reference.

### Boundary Conditions

Standard resistance outlet boundary conditions are employed at truncated arterial boundaries using the following formulation:

1 $$P_{\text{o}} = Q_{\text{o}} \cdot R_{\text{o}} + P_{\text{a}} - \frac{1}{2}\rho \frac{{Q_{o}^{2} }}{{A_{\text{o}}^{2} }}$$

where *P*_*o*_ is the assigned outlet pressure boundary condition, *Q*_o_ the flow rate at the outlet, *R*_o_ the resistance value, *P*_a_ the atrium pressure and *A*_o_ the area of the outlet. The resistance values for neonatal aorta have already been calculated in our previous studies by matching the physiological flow distributions for neonates.^8^ For cerebral outlets, these values are slightly adjusted as reported in Ref. 46 in order to match the physiological pulmonary to systemic flow rate ratio (*Q*_p_/*Q*_s_). The resistance value for each of the cerebral arteries is assumed to be the same: 8 MPa s m^−3^ for RACA, LACA, RMCA, LMCA, RPCA and LPCA.

In the Eq. (1) outlet pressure is calculated based on the flow rate and resistance value of downstream vasculature and organs.

Based on the typical flow rates observed in the clinic, at Aao and MPA a new inlet resistance velocity boundary condition is developed. In this formulation the flow to either the systemic or pulmonary outflow tracts is now determined by the corresponding predefined inlet resistance values, which includes the right/left ventricle pathway and the corresponding outflow trunk valve resistances. Thus, the flow rate is calculated based on the resistance value of the upstream ventricle and pressure at the inlet that reads as;

2 $$Q = \frac{{A_{i}^{2} }}{\rho }\left\{ {\left[ {R_{\text{i}}^{2} - \frac{2\rho }{{A_{\text{i}}^{2} }}(P_{\text{i}} - P_{\text{a}} )} \right]^{1/2} - R_{\text{i}} } \right\}$$

where *Q* is the flow rate at the inlet, *P*_i_ the calculated inlet pressure boundary condition, *R*_i_ the resistance value at the inlet and *A*_i_ the area of the inlet. The plug-flow velocity profile is applied consistently with the standard practice of aortic simulations.

We compared this boundary condition with the standard constant inlet flow boundary conditions. The results did not change for inlet pathway resistance values that are proportional to the inlet cross-sectional areas. However if the resistance values prior to outflow tracts are different, as in various single-ventricle disease states, deviations are recorded. For example, in the case of a ventricle septal defect, this new boundary condition allows the flow exchange between right and left ventricle thus flow splits between aorta and MPA starts to change. The sensitivity of this new inlet boundary scheme is validated by assigning different resistance values for the inlet of the aorta systematically. The increase in aorta inlet pathway resistance decreased the aortic flow and increased MPA flowrate respectively.

### CFD Solver

A commercial CFD solver, FLUENT 15.0 (Ansys, Inc., PA, USA) was adopted for this study. The CFD code was configured to implement a multi-grid artificial compressibility solver for incompressible Newtonian flows, and employs a second-order accurate numerical discretization scheme in space. A steady-state simulation is performed due to the average flow rates at the outlets are found to be sufficient to compare shunt hemodynamics. Also, all Reynolds numbers are below 1500, justifying the use of a laminar flow solver. A diligent mesh density sensitivity analysis followed Ref. 47, based on achieving a relative difference of less than 5% variations in velocity at Dao region just after DA (Supplementary Material, Figure B). Grid sensitivity analysis was conducted using grids of decreasing mesh size (starting with 1.3 mm nodes, to 0.5 mm). For a typical high-density spatial grid with a total of ~1 M fluid nodes, with a grid spacing of 0.7 mm, a simulation time step size of 10^−5^ s in physical time is required to achieve the convergence. Simulations were continued until convergence to 10^−6^ residue. The conservation of mass was ensured of all cases having maximum 10^−8^ L/min difference between inlet and outlet. See the Supplementary Material (Appendix C) for the detailed mesh verification study conducted for both the aorta and the new CoW segments.

## Results

### Pre- and Post-Operative Hemodynamics

**Figure 3:**
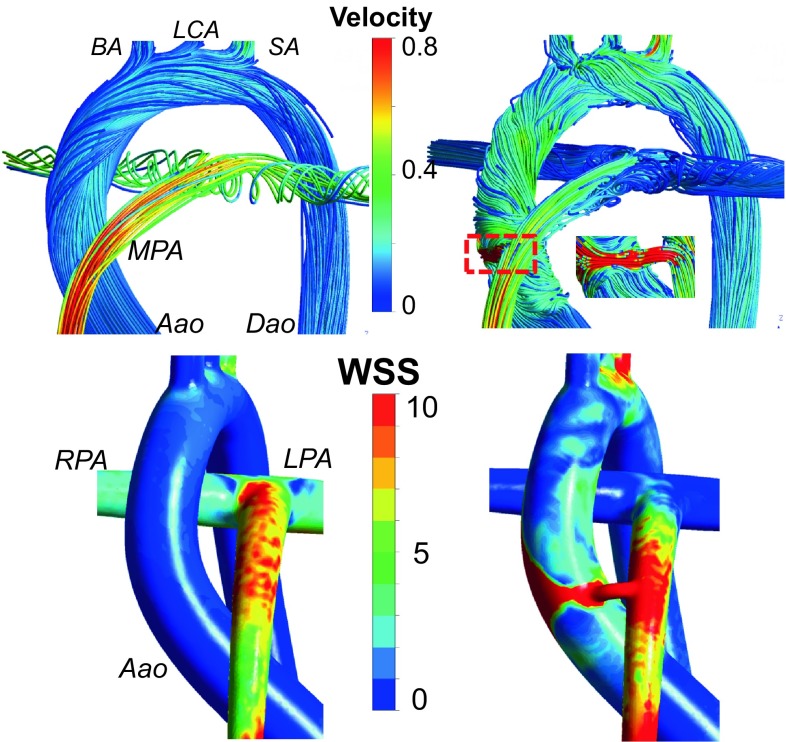
Comparison of the pre-operative model (anatomy before the shunt implantation) (*left column*) with the post-operative direct central shunt configuration (*right column*), for the baseline tetralogy of Fallot (TOF) disease. Percentage differences in head-neck perfusion through the individual aortic arc vessels are labeled at the TOP RIGHT figure. Flow path lines colored with the velocity magnitude are plotted on the top row of the figure and enlarged images of the WSS distributions towards the regions are plotted at the bottom. Results are similar for the pulmonary atresia disease template, which is not shown for brevity. (RPA: Right pulmonary artery, LPA: Left pulmonary artery, Aao: Ascending aorta, MPA: Main pulmonary artery, BA: Brachiocephalic artery, LCA: Left carotid artery, SA: Subclavian artery). Velocity is in m/s and WSS is in N/m^2^. Results are presented for a *Q*_p_/*Q*_s_ of 0.192.

Figure 3 illustrates the flow streamlines and wall shear stress (WSS) distributions for the pre- and post-operative direct shunt configurations. The flow structure and head-neck flow split are altered significantly by the introduction of the shunt. Before the shunt, the entire aortic arch flow was laminar with an average Reynolds number of 300. However, the high velocity gradient around the shunt, due to its small diameter connecting to the large arterial reservoir, altered this condition. The vorticity content is increased particularly at the pulmonary trunk. Likewise, the placement of the shunt increased the WSS levels and its distribution around the aortic arch but decreased it at the pulmonary arteries. There are significant changes in flow rates for all the major vessels as quantified in Table 3, particularly the cerebral arteries. Cerebral flows increase almost two fold after the shunt is implemented. Significant increases are observed for all three shunt configurations with respect to the pre-surgical configuration before the shunt anastomosis.

**Table 3:** Flow rates and the corresponding Reynolds number (in parenthesis) for pre-surgery and the three different shunt configurations (direct, oblique and RPA) investigated for fully-closed ductus arteriosus (DA) state.

Vessel name	Flow rate (LPM)				% Difference wrt direct shunt	
	Pre-Surgery	Direct	Oblique	RPA	Oblique	RPA
Great arterial vessels						
Shunt		0.588 (141)	0.599 (143)	0.643 (148)	2	9
Dao	−0.292	−0.549 (474)	−0.556 (480)	−0.574 (495)	1	5
RPA	−0.416	−0.054 (184)	−0.054 (186)	−0.056 (193)	−4	−23
LPA	−0.416	−0.055 (135)	−0.056 (137)	−0.057 (140)	−5	−23
SA	−0.206	−0.122 (166)	−0.116 (158)	−0.095 (128)	1	4
*Q*_p_/*Q*_s_		0.194	0.183	0.144	−5	−26
Cerebral vessels						
RAC A	0.028	−0.054 (127)	−0.054 (128)	−0.056 (132)	−9	5
LACA	−0.028	0.832 (1089)	0.832 (1089)	0.832 (1089)	1	5
RMCA	−0.029	−0.052 (242)	−0.048 (222)	−0.055 (255)	2	5
LMCA	−0.029	−0.054 (129)	−0.055 (131)	−0.057 (136)	2	4
RPCA	−0.029	−0.122 (163)	−0.116 (156)	−0.094 (126)	1	5
LPCA	−0.029	−0.054 (145)	−0.054 (146)	−0.056 (153)	1	5
*Q*_c_/*Q*_CO_		0.215	0.214	0.225	0	5

Last two columns summarize the differences in flow rates for shunt configurations with respect to the direct (central) shunt. Only the vessels with significant differences between the surgical configurations are included. Negative flow rate values represent outlet flows. *Q*_p_/*Q*_s_: Ratio of pulmonary flow to systemic flow. *Q*_c_/*Q*_CO_: Ratio of total cerebral flow to cardiac output

### Comparison of Shunt Configurations

**Figure 4:**
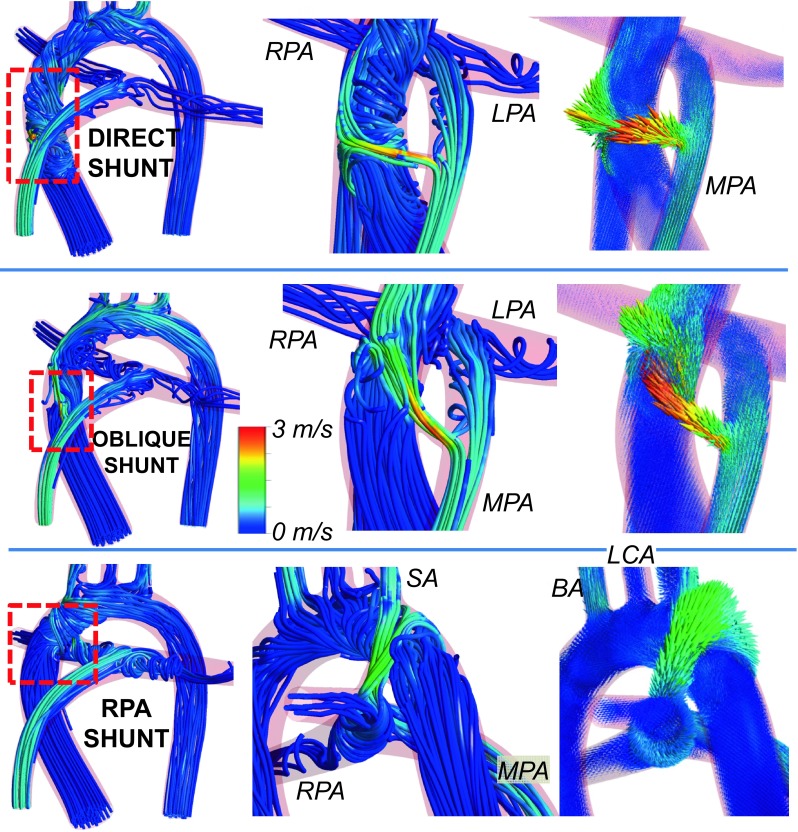
Comparison of shunt configurations for the full model of tetralogy of Fallot (TOF) on aortic arch and pulmonary arteries. Figures on the left and at the middle show flow path lines colored with the velocity magnitude, while figures on the right show velocity vectors. The entire aortic region is displayed on the left, while the middle and right columns focused on shunt regions. Results are presented for the central direct, central oblique and RPA shunts from top to bottom, respectively. (MPA: Main pulmonary artery, RPA: Right pulmonary artery, LPA: Left pulmonary artery, BA: Brachiocephalic artery, LCA: Left carotid artery, SA: Subclavian artery.). Results are presented for high pulmonary vascular resistance (i.e., *Q*_p_/*Q*_s_ is between 0.144 and 0.192).

In Fig. 4, the post-operative flow structures and streamlines are plotted for all three shunt configurations. For the direct and RPA shunts the corresponding flow streamlines spiral in the ascending aorta influencing the head-neck flow split and WSS distribution. For the oblique shunt, the flow in the ascending aorta is relatively laminar. According to Fig. 4, for RPA shunt the flow produces high vorticity in both pulmonary arteries. Vorticity in pulmonary arteries is lower for oblique shunt configuration. Direct shunt produces almost laminar flow in both pulmonary arteries.

The average flow splits at the aortic arch and cerebral arteries for all 3 models are summarized in Table 3. Flow rates at the pulmonary arteries change 5% between the central direct shunt and central oblique shunt configurations and 23% between central direct shunt and RPA shunt configurations. The right and left pulmonary artery flow rates are not altered significantly for the different shunt configurations as the symmetric flow condition is maintained. At cerebral arteries shunt type caused about 2 and 5% differences in flow rate for the central oblique shunt and RPA shunt configurations, respectively. Whereas the flow splits between right and left cerebral arteries are not symmetric for the oblique shunt configuration (see Table 3). Likewise, the trans-shunt flow changes up to 10%, between the direct (0.588 LPM) and the RPA shunt (0.643 LPM). *Q*_p_/*Q*_s_ for shunt configurations are also calculated and found to be 5 and 26% different for oblique and RPA shunts, respectively. These results suggest that the acute post-operative hemodynamic condition depends on the shunt configuration, and shunt configurations change the flow rates substantially.

Results presented in Table 3 correspond to a patient with a very high pulmonary vascular resistance value of 8 MPa s m^−3^ (for details see Supplementary Material, Appendix B). This resistance results in a low *Q*_p_/*Q*_s_. To illustrate the performance at a higher *Q*_p_/*Q*_s_ and thus lower pulmonary vascular resistance, we performed simulations with a resistance value of 3 MPa s m^−3^ for both RPA and LPA outlet boundary conditions. Results for these cases are presented in Table 4.

**Table 4:** Flow rates and the corresponding Reynolds number (in parenthesis) for the three different shunt configurations (direct, oblique and RPA) investigated for fully-closed ductus arteriosus (DA) state with low pulmonary resistance value.

Vessel name	Flow rate (LPM)			% Difference wrt direct shunt	
	Direct	Oblique	RPA	Oblique	RPA
Great arterial vessels					
Shunt	−0.392 (101)	−0.379 (97)	−0.569 (146)	−3	45
Dao	−0.241 (208)	−0.255 (221)	−0.193 (166)	6	−20
RPA	−0.258 (341)	−0.262 (347)	−0.299 (395)	2	16
LPA	−0.352 (470)	−0.335 (447)	−0.488 (651)	−5	39
SA	−0.169 (298)	−0.178 (313)	−0.135 (238)	5	−20
*Q*_p_/*Q*_s_	0.683	0.659	1.098	−4	61
Cerebral vessels					
RACA	−0.070 (329)	−0.051 (237)	−0.055 (258)	−28	−22
LACA	−0.079 (272)	−0.078 (269)	−0.062 (216)	−1	−21
RMCA	−0.084 (200)	−0.087 (209)	−0.068 (163)	5	−19
LMCA	−0.086 (213)	−0.091 (224)	−0.070 (173)	5	−19
RPCA	−0.082 (223)	−0.082 (224)	−0.066 (181)	1	−19
LPCA	−0.082 (194)	−0.084 (198)	−0.066 (157)	2	−19
*Q*_c_/*Q*_CO_	−0.321	−0.315	−0.258	−2	−20

Last two columns summarize the differences in flow rates for shunt configurations with respect to the direct (central) shunt. Only the vessels with significant differences between the surgical configurations are included. Negative flow rate values represent outlet flows. *Q*_p_/*Q*_s_: Ratio of pulmonary flow to systemic flow. *Q*_c_/*Q*_CO_: Ratio of total cerebral flow to cardiac output

**Figure 5:**
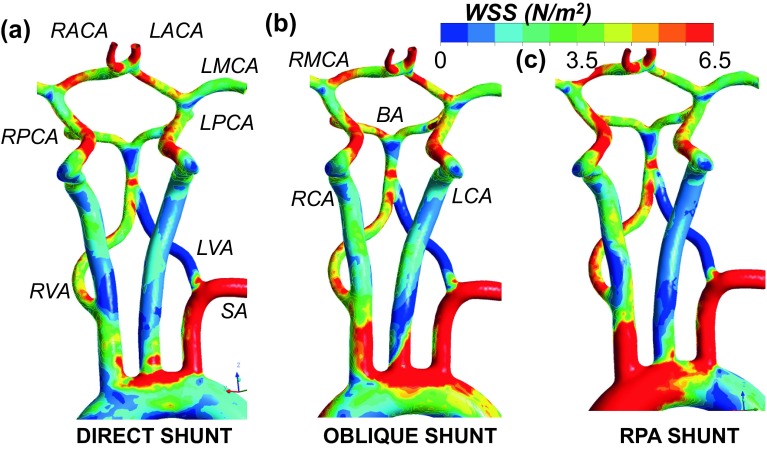
Hemodynamic comparison at the neck segment for the three shunt configurations studied in the present manuscript; direct, oblique and RPA shunt configurations. Colored figures display the WSS distributions. (SA: Subclavian artery, RVA: Right vertebral artery, LVA: Left vertebral artery, RCA: Right carotid artery, LCA: Left carotid artery, BA: Basilar artery, RACA: Right anterior cerebral artery, LACA: Left anterior cerebral artery, RMCA: Right middle cerebral artery, LMCA: Left middle cerebral artery, RPCA: Right posterior cerebral artery, LPCA: Left posterior cerebral artery.) Results are presented for high pulmonary vascular resistance (i.e., *Q*_p_/*Q*_s_ is between 0.144 and 0.192).

**Figure 6:**
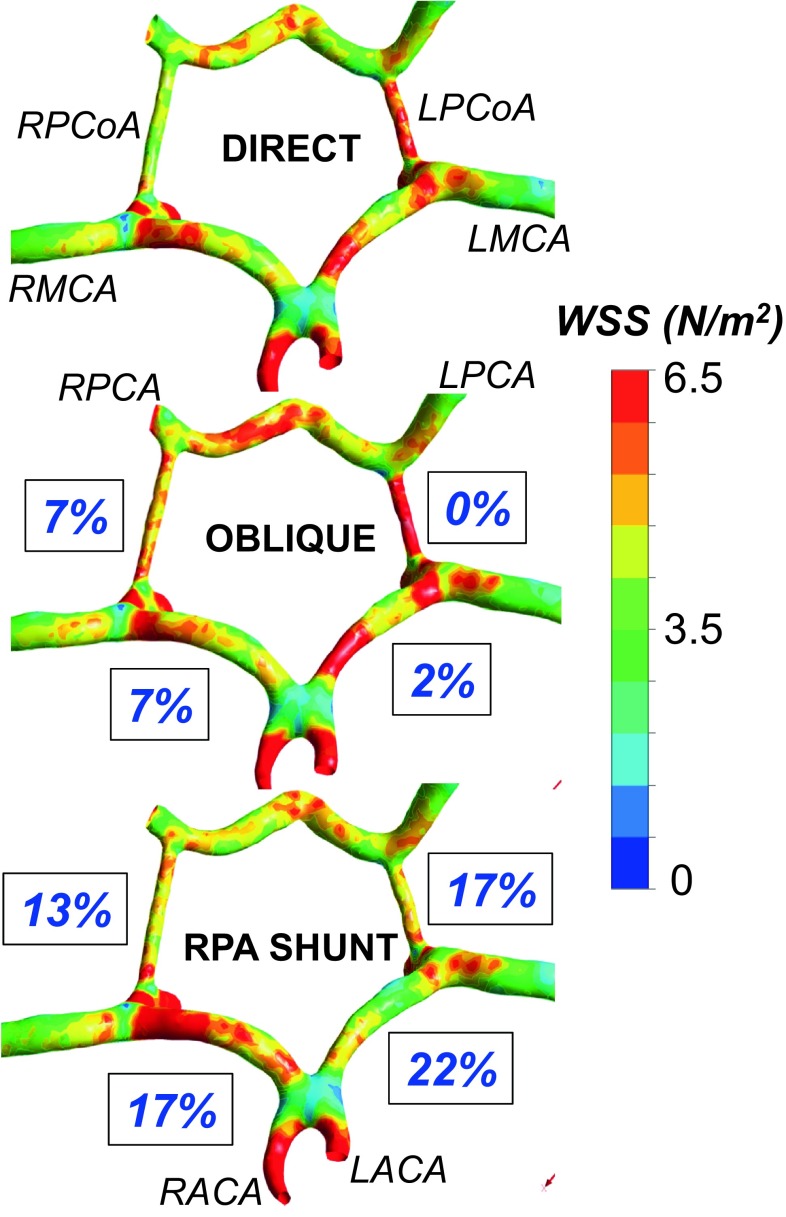
Post-operative flow differences and wall shear stress (WSS) distribution at the circle of Willis (CoW) due to different surgical shunt types; short, oblique and RPA. The percentage values labeled on each cerebral artery correspond to the flow rate differences due to shunt type relative to the direct shunt configuration (TOP figure). (RACA: Right anterior cerebral artery, LACA: Left anterior cerebral artery, RMCA: Right middle cerebral artery, LMCA: Left middle cerebral artery, RPCA: Right posterior cerebral artery, LPCA: Left posterior cerebral artery, RPCoA: Right posterior communicating artery, LPCoA: Left posterior communicating artery.). Results are presented for high pulmonary vascular resistance (i.e., *Q*_p_/*Q*_s_ is between 0.144 and 0.192).

Figure 5 illustrates the WSS levels for the direct, oblique and RPA shunts at neck and head arteries. Likewise, Fig. 6 provides the WSS changes specifically at the CoW region. The WSS is about 20% higher for the RPA shunt configuration compared to the direct shunt configuration, especially at the aortic arch region. The shunt anastomosis feature higher WSS distribution compared to its periphery. Maximum WSS values are 118, 107 and 48 Pa for direct, oblique and RPA shunt configurations, respectively. Direct shunt configuration produces the highest WSS at the shunt region, although it does not have the highest shunt flow rate. Geometry of the shunt and its anastomosis are the determinants of WSS distribution in addition to the flow magnitude, as demonstrated earlier.^12^

Finally, the flow rate through the internal arteries of CoW region is computed and compared for all three shunt configurations. The internal arteries that are particularly important for intra-operative diagnosis include RPCoA, LPCoA, and the connecting arteries of RMCA-RPCA and LMCA-LPCA. These internal arteries are important as they regulate the blood flow to the cerebral artery through the afferent arteries. Figure 6 presents the relative flow differences in internal CoW arteries for oblique and RPA shunt configurations compared to direct shunt configuration. The shunt configurations affect flow distribution in the head and neck arteries and can challenge a balanced regional brain perfusion that can be taken into account in pre-surgical planning. The differences in flow splits start at the connecting cerebral arterial level influencing the downstream cerebral vascular perfusion.

### Effect of Ductus Arteriosus Constriction on Shunt Hemodynamics

Although the results of the present study represent neonatal patients without a DA, some patients have a functioning DA (naturally or deliberately through a stent). The fully open DA model corresponds to the hybrid reduced (less) invasive interventional therapy achieved through a stented DA with banded PAs. This configuration also represents the early post-operative state observed in a limited number of 1st stage patients. Therefore, in this section hemodynamics of shunt configurations with closed DA (“Comparison of Shunt Configurations” section) are compared with the open DA state. Simulations are completed for all three shunt configurations, but for brevity only the direct shunt configuration results are presented here, as the conclusions were similar. It is observed that the flow through the pulmonary arteries changes 42% when a persistent DA exists. The cerebral flow changes only 5%, while the shunt flow increases sixfold with the DA.

Flow distributions at all artery outlets and the *Q*_p_/*Q*_s_ stays almost constant for all three shunt configurations while DA is active. This is due to the high peripheral resistance values of the neonatal patient, which determines the flow rates, consistent with our previous study.^8^ Interestingly, the flow passing through shunt and PDA depends on the shunt configuration. The trans-shunt flowrate is 21% higher for central oblique shunt compared to central direct shunt when DA is patent. Likewise, the flow rate at the RPA shunt is 53% higher than that of central direct shunt, resulting in improved shunt performance for TOF (fully-open MPA model). Flow rate at the PDA decreases 3 and 8% (0.02 LPM and 0.03 LPM) when compared to central direct shunt configuration for central oblique and RPA shunt configurations, respectively. Those decreases in flow rates balance the shunt flows so that the flow convected to the pulmonary arteries stay relatively constant.

Finally, the flow rates at the major interior cerebral branches depend on the shunt configuration. For example, the artery connecting the LMCA and anterior arteries has 3% higher flow rate for RPA shunt configuration compared to central direct shunt configuration for the TOF disease model.

## Discussion

During the last decade, neonatal surgical repair of TOF resulted in minimal mortality, and the present focus shifted towards achieving the best late-functional outcome.^11^ It is hypothesized that the optimal shunt hemodynamics is critical for improved quality of life. As our morphometric study demonstrated, shunt placement will balance the post-operative *Q*_p_/*Q*_s_ and minimize pulmonary hypertension^1^,^21^,^49^,^51^,^52^ in a shunt configuration dependent manner. Local shunt flow performance, particularly WSS, is known to influence the shunt patency, and can vary as much as 33% between the different shunt configurations. Higher WSS distributions will decrease the graft life due to high friction at the material surface. Likewise, analyzing the WSS distribution at the head-neck, neck and cerebral arteries for direct and RPA shunts indicates higher WSS at distal LCA and vertebral artery anastomosis for RPA shunt compared to the direct-shunt configuration (Fig. 5).

We have simulated two different pulmonary vascular resistance states: with high and low values (Tables 3 and 4). For the high pulmonary vascular resistance state: Shunt configuration can also cause substantial changes in the total PA flow leading to 26% changes in *Q*_p_/*Q*_s_ for the same shunt diameter. However, once the peripheral pulmonary vascular resistances at RPA and LPA are fixed there is no significant preferential flow direction between RPA and LPA, for all shunt configurations studied in this work. The DA patency (naturally or after the stenting operation) and disease severity does not alter these flow regimes. Therefore, realistic measurements of pulmonary vascular resistances are critical in predicting the pulmonary flow preference. In our model, we intentionally kept RPA and LPA geometries at the same diameter in symmetric shape, which resulted in the same great artery resistance at uniform flow conditions. Any change in geometry, peripheral or branch resistance (e.g., due to complex flow patterns in one branch, see Figs. 3 and 4) could cause a difference in LPA/RPA ratio. Also, larger shunt or pulmonary artery diameter could increase the *Q*_p_/*Q*_s_. For the high pulmonary vascular resistance state: *Q*_p_/*Q*_s_ can change up to 61% for the same shunt diameter while RPA and LPA flow rates also differ significantly within the same shunt configuration (up to 63%). Thus, the pulmonary vascular resistance, besides shunt configuration, has a substantial effect on the arterial flow splits.

Considering the complex recirculation regions (vorticity), the direct shunt is the most laminar flow among the shunt configurations studied. Vorticity is important in terms of energy loss and blood damage and should be avoided.^15^,^35^,^56^ Since the shunt position affects the formation of vortices, it is also expected to affect flow split at the artery outlets and WSS at the root of the head neck arteries. Therefore, existence of vorticity should also be taken into consideration in terms of surgery performance.

Present results illustrate an important function of DA as a balancing vessel as there are substantial differences between cases with and without DA. For an active and functioning DA, the influence of shunt configuration on hemodynamic balance is found to be minimal. For an open DA, the shunt diameter and configuration cannot control the *Q*_p_/*Q*_s_ and achieve hemodynamic stability. Regardless of the shunt type, all arterial flow splits will remain the same. Shunt size does not allow enough blood flow to maintain the same flow distribution. In contrast, when the DA is closed the head-neck flow distributions and *Q*_p_/*Q*_s_ become highly dependent on shunt configurations in addition to the resistances of peripheral arterial beds. While the Open-DA configuration can be employed in hybrid repair, the closed DA case is more common in pediatric patients and achieves better circulatory control since it is ligated by the surgeon or tends to vanish naturally after birth.

In an earlier study, through an idealized parametric computational model of hypoplastic left heart syndrome, Migliavacca *et al.* 29 calculated pressure drops for straight and blunt shunt configurations that resemble the direct and oblique shunts of the present study, to be ~30 and ~26 mmHg respectively. Even though the type of disease is considerably different, the pressure drop values are in agreement with the present computations. As such, the pressure drops for all flow rates are higher for straight shunts compared to the blunt shunts. In terms of higher pulmonary perfusion and lower pressure drops, the surgeon may prefer the direct shunt during surgery.

Neurodevelopmental delays in CHD patients are common and highly variable.^17^,^34^ Present results demonstrate that the placement of the surgical shunt alters the head-neck flow split and the acute hemodynamic balance of the cerebral circulation system. The differences in flow rates in cerebral arteries indicate different perfusion rates at the vital brain sections. Whether this finding might have major physiological consequences or be associated with the poor post-operative neurodevelopment outcome of 1st stage surgeries should prompt further investigations. Still, it would be wise to consider cerebellum blood perfusion as a new performance parameter that can easily be calculated in 1st stage computational pre-surgical planning. This enables an estimate of the flow changes in the brain after shunt surgery. Particularly the role of CoW to redistribute the cerebral flow after the acute shunt placement is an important clinical factor and has not been emphasized in the literature to our knowledge. Knowledge of the detailed post-operative 3D cerebral perfusion map could lead to optimal neurodevelopment.

Patient-specific computational fluid dynamics evolved to be a standard tool for simulating the hemodynamic performance of pediatric cardiovascular shunts, reducing the need for *in vitro* tests as well as complex animal experiments.^42^ While most steps of the patient-specific analysis methodology including the MRI scanning, segmentation, volume generation, mesh discretization and visualization has matured,^42^ as our study indicates, the predictive capability of realistic boundary conditions representing the peripheral circulation needs further emphasis. Particularly the inclusion of major cerebral vessels undertaken in the present work is a step towards this objective. We showed that the standard resistance boundary conditions attached to the truncated head-neck vessels outlets representing the cerebral circulation is not adequate for predicting flow-splits as well as the local flow properties such as streamlines, WSS and pressure distribution (See Supplementary Material Appendix B for details). According to our results, the standard CFD model without the head-neck and cerebral arteries overestimates the flow passing through the shunt and underestimates the DA flow. Likewise, inclusion of the full cerebral system substantially changes the flow distribution and shifts the flow balance at the head-neck arteries. This finding is more critical for the smaller size neonatal aortic arch system compared to a *mature* aortic arch, since for the later the peripheral vascular resistance values are significantly lower. As our results demonstrate, the hemodynamic shunt analysis cannot be localized to the shunt region alone. The entire cardiovascular circulation system, including the natural shunt of DA, if it exists, must be considered for precise surgical decision-making.^42^ Finally, the addition of 3D cerebral arteries to the CFD domain will not eliminate the utility of lumped parameter model boundary conditions as they will still be needed for the rest of the vasculature.

## Limitations

The present study is a pilot investigation that focused on diseased type and shunt configurations, which will be expanded through larger shunt sizes, surgeon-specific shunt configurations and parametric pulmonary arterial diameters,^45^ including patient-specific anatomical cases as they become available.^44^ Computational results correspond to the time-averaged hemodynamics and exclude the deformation of the artery as well as the non-Newtonian effects. The latter parameter is potentially important, but its effect is shadowed due to the high variability of pediatric blood and so would not influence our comparative results. As in most arterial hemodynamic applications, for aortic flows the use of compliant models (for the deformation of the artery compared to aortic root rotation) alone does not bring much improvement on the accuracy of results over simpler and computationally more efficient rigid models.^20^,^25^,^31^,^36^,^41^ We utilized a patient-specific cerebral arterial anatomy but developed a realistic arch reconstruction through the diligent input from several experienced clinicians on this integrated model (model development is summarized in Supplementary Material, Appendices A and B). Idealized aortic arch and neck arteries can cause some deviations from the patient-specific results, but this effect is limited since, in the present study, the shunt configurations are compared to each other using the same baseline geometry.

Our results clearly illustrate that the predictive surgical planning simulations require the use of an accurate downstream patient-specific cerebral geometry. Complexity of the cerebral arterial system is a major challenge for the present study and needs to be revised and improved in future models. For example, an incomplete CoW is common for neonates and congenital heart patients, which should influence the reported flow splits. Still, the comparative values of present results should be valid to a certain degree. The physiologically realistic geometry and boundary conditions are critical for replicating the physiological results, even if it is challenging to obtain accurate measurements and data needed for modeling purposes in infants and small babies.^22^,^42^,^43^ Likewise, the effects of disease-specific shunt configurations and anastomosis location on cerebral and coronary flow are all important considerations for the surgical decision-making process.

Our downstream boundary conditions are not fully multi-scale, still the present boundary conditions are indeed the “lumped” versions of more detailed multi-scale boundary conditions, thus both simulate the same physical behavior. Our manuscript demonstrated an important weakness of these schemes, applicable both for lumped or multiscale; the 3D cerebral system geometry in simulations is critical for accurate estimation of changes in especially WSS and 3D flow characteristics: secondary flow and vorticity as highlighted in the original Appendix material (Page 2, Section B).

## Conclusions

The present manuscript explored alternative shunt configurations that have potential for improved peripheral blood flow split and local hemodynamics. Quantitative information on cerebral hemodynamics and perfusion are provided, which are critical for CHD patients. Our study showed that the RPA shunt has slightly better cerebral perfusion for TOF. Furthermore, a persistent ductal communication between the systemic and pulmonary arteries suppresses the influence of surgical shunt and results in poor flow split control. When the ductus arteriosus is fully ligated, all three clinically shunt configurations result in significant differences in flow distributions and local hemodynamics. Most importantly, major differences observed in cerebral blood flows prompted the requirement for detailed future studies on neonatal cerebral perfusion of CHDs. The shunt configuration has a very limited, almost no, effect on flow splits while the DA is open and is critical for flow control when the DA is closed. In addition to the shunt configuration, our computations indicated that neonatal arterial hemodynamics is also influenced by the pulmonary vascular resistance severity and should be taken into consideration during the 1st stage shunt planning (for example, for high pulmonary resistance case, direct shunt has 26% higher pulmonary perfusion with respect to RPA shunt while for low pulmonary resistance case RPA shunt has 61% higher pulmonary perfusion with respect to direct shunt). Surgeons can prefer direct shunt in terms of higher pulmonary perfusion (23% with respect to RPA shunt perfusion) and lower pressure drop, even though it has 5% lower cerebral perfusion in the case of TOF in case of low pulmonary vascular resistance. Current practice in hemodynamic modeling, including the lumped parameter system models, is to consider aortic arch manifold vessel as central, and to treat the neck and cerebral arteries as a lumped network or as a *truncated* constant pressure boundary condition. As the present study illustrates, if such truncated boundary conditions are utilized, the results might be misleading.

## Electronic supplementary material

Below is the link to the electronic supplementary material.

Supplementary material 1 (DOCX 1308 kb)
