# Supplementary material for: Tetralogy of Fallot Surgical Repair: Shunt Configurations, Ductus Arteriosus and the Circle of Willis
Source: Cardiovasc Eng Technol. 2017 Apr 5;8(2):107–19. doi: 10.1007/s13239-017-0302-5 (PMC5446850; doi:10.1007/s13239-017-0302-5)
Supplement: Supplementary file 1 — Supplementary material 1 (DOCX 1308 kb) [file 13239_2017_302_MOESM1_ESM.docx]

**Supplementary Material**

**Table of Contents:**

Appendix A: Idealized model history and validation of the cerebral segment

Appendix B: Improvements in predictive accuracy due to the use of full cerebral arterial segment

Appendix C: Mesh Generation and Convergence Analysis

References

Figures

**Appendix A: Idealized model history and validation of the cerebral segment**

For both the newborn and congenital heart patients, the patient specific cardiovascular anatomical reconstructions are very rare in the literature. This is partially due to the fact that the high-resolution computed tomography (CT) scans for research purposes involve ethical issues for the newborn babies and the magnetic resonance imaging (MRI) has resolution challenges and risks in sedation for this age group. Thus an established realistic idealized neonatal arterial geometry is used in the present study. Pekkan et al. have generated the present idealized neonatal aortic arch model using parametric computer aided design tools (eg. Proengineer, solid works, etc) [1]. Its anatomical dimensions and orientations have been selected based on literature [2-4] and confirmed through the interviews with 3 experienced pediatric surgeons and radiologists by matching normal vessel diameters and shapes. In order to faithfully represent the great-vessel architecture of common congenital heart defects (CHD), the normal neonatal aortic arch model has been further modified through the same experience-based quantitative approach [5, 6]. The selected common CHD templates include the hypoplastic left heart syndrome (HLHS), tetralogy of Fallot and pulmonary atresia. In addition to the human newborn clinical flow measurements that are summarized recently by our group [7] the computational method used in the present and in the previous studies [8] have been further validated against *in vitro* experiments [9] and *in vivo* neonatal piglet model pressure data which is an established animal model for the neonatal cardiopulmonary bypass [1]. De Zelicourt et al [8] have studied the HLHS template through the same baseline geometric model and computational method which were validated by Pekkan et al [1] and Wang et al [9]. Menon et al [10] have studied the performance of jet wakes emanating from state-of-the-art pediatric cannula tips using the same aortic arch geometric template and validated computational fluid dynamics (CFD) results qualitatively with time resolved flow visualization and quantitatively with particle image velocimetry (PIV). Thus, an extensively validated neonatal arch geometry is used in the present study as the base model (model without head-neck and cerebral arteries).

In the present manuscript the neonatal aortic arch model is further improved by the addition of cerebral vessels and the head-neck arterial system. The diameter of the aorta is 12 mm. The diameters of head-neck and cerebral arteries used in the present study are compared to the angiographic measurements of children from literature [11] and are found to be within the standard deviation. For example, Arat et al [11] reports the range of diameters for the middle cerebral artery 1.90 and 3.20 mm, for the anterior cerebral artery 1.20 and 3.00 mm and for internal carotid artery at cavernous segment 2.60 and 4.70 mm for children between the age of 6 and 72 months. In the present study we employed 2.75 mm, 2.00 mm and 4.10 mm for the diameters of the corresponding arteries, respectively.

**Appendix B: Improvements in the predictive accuracy due to the use of full cerebral arterial segment**

The full relative values of the peripheral systemic vascular resistances downstream of the neonatal aortic arch system prompted the present verification study. From clinical catheterization measurements, we observed that, unlike the adult circulation system, the peripheral blood flow resistances downstream of the neonatal aortic arch are very high compared to the vascular resistance of the shunted aortic arch great vessels. These extremely high peripheral resistance values decrease the sensitivity of great vessel hemodynamics to the shunt diameter and configuration, unless the detailed cerebral vessel circuit is included in the CFD model. Thus the inclusion of the full cerebral arterial segment into our model allowed us to investigate the detailed acute blood flow changes, wall shear stress and pressure distribution throughout the three dimensional (3D) cerebral arteries which is important for post-op neurodevelopmental performance and surgical planning.

To prove the aforementioned importance of the cerebral arteries, CFD simulations were run for two different model configurations: *full geometry model that includes head-neck and cerebral arteries with Circle of Willis* (w CoW) and *aortic arch only geometry model without the Circle of Willis* (w/o CoW). The model w/o CoW consists of totally 2 inlets and 5 outlets; 2 at pulmonary arteries and 3 at neck arteries (see Figure A). This model has resistance boundary conditions at the outlets; Dao, RPA, LPA, BA, LCA, and SA. The model w CoW consists of totally 2 inlets and 10 outlets; 6 at cerebral arteries, 4 at aortic arch (Main Text, Figure 1), having resistance boundary conditions at; Dao, RPA; LPA, SA, RACA, LACA, RMCA, LMCA, RPCA, and LPCA. Both models are identical except the additional cerebral geometry; they both have the same inlet boundary conditions (same flow rates) at Aao and MPA. We tested the effect of including the full-cerebral arterial system using the direct shunt and tetralogy of Fallot (toF) disease template: Cases 41, and 42 (Main Text, Table 2).

The resistance values for the isolated aorta geometry (model w/o CoW) have already been calculated in our previous study by matching the physiological flow distributions for neonates [8]. They are 2.4, 5.9, 3.9, 1.2, and 2.7 MPa.s.m^-3^ for BA, LCA, SA, Dao and RPA, and LPA, respectively. For the model w CoW, the calculation of resistance values at cerebral outlets was done in our previously published [12] and present study by matching approximately the same flow distributions at the aortic arch outlets (Dao, LPA and RPA) and maintaining the physiological pulmonary to systemic flow (Q_P_/Q_S_) ratio. Thus the boundary conditions are made identical for both models and allowed us to compare the core aortic flow fields.

Our results indicated that inclusion of cerebral arteries to the aortic arch system significantly alters the great vessel hemodynamics in the aortic arch. To illustrate this difference qualitatively, the streamlines for both models are plotted in Figure A. Especially at aortic isthmus, where the DA is connected to Dao (i.e. downstream of DA), flow path lines of the full-model resulted complex flow structures influencing the head-neck flow split where percent flow split differences are labeled for BA, LCA, SA in Figure A. The CFD model w/o CoW does not capture this complex flow regime. Figure A also shows the WSS distribution throughout the aortic arch for standard resistance boundary conditions (model w/o CoW) vs. with the full cerebral system (model w CoW). While the spatially averaged difference in maximum WSS is about 7% (model w/o CoW has lower WSS values), there are significant local hot-spot differences in WSS proximal to the surgical shunt and the natural ductal shunt, reaching 35%, where the model w CoW has higher WSS values, as given in Figure A. These differences are also reflected to the DA and shunt flow rates where flow passing through the DA is about 0.475 L/min for model w/o CoW and 0.566 L/min for model w CoW resulting a difference of 19%. Trans-shunt flow is also affected similarly; 0.113 L/min for model w/o CoW and 0.085 L/min for model w CoW, ~30% difference.

Since the resistance boundary conditions with realistic values are used in the computational model, the aortic root pressure represents an approximate index for the coronary arterial perfusion. This pressure found to be different for two models; 82 and 64 mmHg for models w/o CoW and w CoW, respectively. Inclusion of the cerebral system decreases the aortic root pressure 25% which is also substantial. As such, the model with full geometry (model w CoW) has 5% higher maximum velocity magnitude than that of the isolated aortic arch model (model w/o Cow), 0.83 m/s and 0.79 m/s, respectively. Therefore, all remaining results in this study are obtained using the full arterial model (model w CoW) that includes the cerebral system and the CoW.

In fact, one can get any flow distribution at the aortic outlets by adjusting the distal resistance values, for both models (full and truncated). However the objective is to perform “surgical planning” with these resistance boundary condition sets; i.e. we like to implement different shunt configurations and analyze their acute hemodynamic effects. When we introduce the same shunt, to both models (full and truncated with matched outflows), each model will result a different acute flow distribution in the aorta. The full Circle of Willis model will provide a more physiological downstream great vessel characteristics.

**Appendix C: Mesh Generation and Convergence Analysis**

The Ansys Mesher 15.0 (Ansys, Inc., PA, USA) grid generation package was used to generate the 3D mesh. Assessment of the mesh quality was defined as devoid of high cell skewness, as there is a known relationship between mesh quality, simulation convergence, and convergence time [13]. A diligent mesh density sensitivity analysis followed reference [14] based on achieving a relative difference of less than 5% variations in velocity at Dao region just after DA (Figure B). Mesh sensitivity studies of the isolated aortic arch domain have been also performed in our earlier studies [8, 15] but verified again in the present work. Likewise we also completed auxiliary tests with the cerebral arterial system alone and ensured local mesh convergence [12]. The combined model (w CoW) geometry was discretized using curvature and radius dependent adaptive mesh elements. Grid sensitivity analysis was conducted using grids of decreasing mesh size (starting with 1.3 mm nodes, to 0.5 mm). Regular mesh size (0.7 mm) was selected for meshing the whole domain and this mesh size was used in all simulations. The element density used at cerebral arteries and aortic arch is kept the same. The element size versus total number of elements of the coupled model (model w CoW) is presented in Figure B. An additional grid sensitivity analysis was conducted for local cerebral artery mesh (at Basilar Artery region) and the resulting mesh size was found to be the same with aortic arch (0.7 mm). The type of the elements used in all meshes is 3D 10-noded (quadratic) tetrahedral elements. Skewness is less than 0.83 for all mesh elements.

**References**

[1] K. Pekkan *et al.*, "In Vitro Hemodynamic Investigation of the Embryonic Aortic Arch at Late Gestation," *Journal of biomechanics,* vol. 41, no. 8, p. 10.1016/j.jbiomech.2008.03.013, 2008.

[2] R. Achiron, S. Zimand, J. Hegesh, S. Lipitz, Y. Zalel, and Z. Rotstein, "Fetal aortic arch measurements between 14 and 38 weeks' gestation: in-utero ultrasonographic study," (in eng), *Ultrasound Obstet Gynecol,* vol. 15, no. 3, pp. 226-30, Mar 2000.

[3] W. Long, *Fetal and Neonatal Cardiology*. Philadelphia: Saunders, 1990.

[4] G. Mielke and N. Benda, "Reference ranges for two-dimensional echocardiographic examination of the fetal ductus arteriosus," (in eng), *Ultrasound Obstet Gynecol,* vol. 15, no. 3, pp. 219-25, Mar 2000.

[5] A. M. Ilbawi, D. E. Spicer, S. Bharati, A. Cook, and R. H. Anderson, "Morphologic study of the ascending aorta and aortic arch in hypoplastic left hearts: surgical implications," (in eng), *J Thorac Cardiovasc Surg,* vol. 134, no. 1, pp. 99-105, Jul 2007.

[6] S. F. Wong, C. Ward, A. Lee-Tannock, S. Le, and F. Y. Chan, "Pulmonary artery/aorta ratio in simple screening for fetal outflow tract abnormalities during the second trimester," (in eng), *Ultrasound Obstet Gynecol,* vol. 30, no. 3, pp. 275-80, Sep 2007.

[7] M. B. Yigit, W. J. Kowalski, D. J. Hutchon, and K. Pekkan, "Transition from fetal to neonatal circulation: Modeling the effect of umbilical cord clamping," (in eng), *J Biomech,* vol. 48, no. 9, pp. 1662-70, Jun 25 2015.

[8] D. de Zélicourt, P. Jung, M. Horner, K. Pekkan, K. R. Kanter, and A. P. Yoganathan, "Cannulation Strategy for Aortic Arch Reconstruction Using Deep Hypothermic Circulatory Arrest," *The Annals of Thoracic Surgery,* vol. 94, no. 2, pp. 614-620, 8// 2012.

[9] C. Wang *et al.*, "Progress in the CFD modeling of flow instabilities in anatomical total cavopulmonary connections," (in eng), *Ann Biomed Eng,* vol. 35, no. 11, pp. 1840-56, Nov 2007.

[10] P. G. Menon, J. F. Antaki, A. Undar, and K. Pekkan, "Aortic outflow cannula tip design and orientation impacts cerebral perfusion during pediatric cardiopulmonary bypass procedures," (in eng), *Ann Biomed Eng,* vol. 41, no. 12, pp. 2588-602, Dec 2013.

[11] Y. O. Arat, A. Arat, and K. Aydin, "Angiographic Morphometry of Internal Carotid Artery Circulation in Turkish Children," (in eng), *Turk Neurosurg,* vol. 25, no. 4, pp. 608-16, 2015.

[12] S. Piskin, A. Undar, and K. Pekkan, "Computational Modeling of Neonatal Cardiopulmonary Bypass Hemodynamics With Full Circle of Willis Anatomy," (in Eng), *Artif Organs,* 2015.

[13] L. Antiga, B. Ene-Iordache, L. Caverni, G. P. Cornalba, and A. Remuzzi, "Geometric reconstruction for computational mesh generation of arterial bifurcations from CT angiography," *Computerized Medical Imaging and Graphics,* vol. 26, pp. 227-235, 2002.

[14] S. Prakash and C. R. Ethier, "Requirements for mesh resolution in 3D computational hemodynamics," *Journal of Biomechanical Engineering,* vol. 123, pp. 134-144, 2001.

[15] K. Pekkan *et al.*, "Neonatal aortic arch hemodynamics and perfusion during cardiopulmonary bypass," (in eng), *J Biomech Eng,* vol. 130, no. 6, p. 061012, Dec 2008.

**Figures**


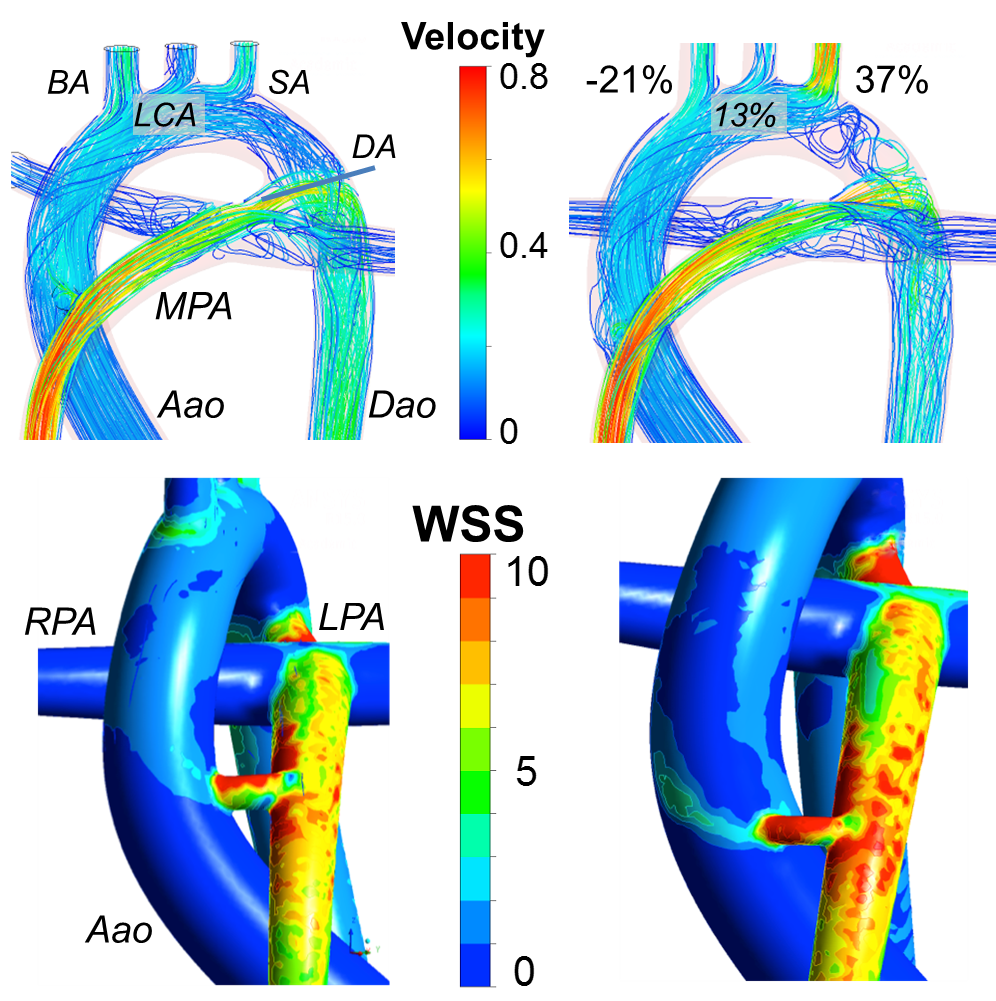


**Figure A**: Comparison of model without neck and cerebral arteries (*LEFT COLUMN*) with the full model (*RIGHT COLUMN*) for tetralogy of Fallot simulations. Percentage differences in head-neck perfusion through the individual aortic arc vessels are labeled on TOP RIGHT figure. Flow path lines colored with velocity magnitude are plotted on the top row of the figure and WSS distributions with zoomed shunt regions are plotted at the bottom. Results are similar for the pulmonary atresia template and not shown for brevity. (RPA: right pulmonary artery, LPA: left pulmonary artery, Aao: Ascending aorta, MPA: Main pulmonary artery, BA: brachiocephalic artery, LCA: left carotid artery, SA: subclavian artery). Velocity is in m/s and WSS is in N/m^2^.

**
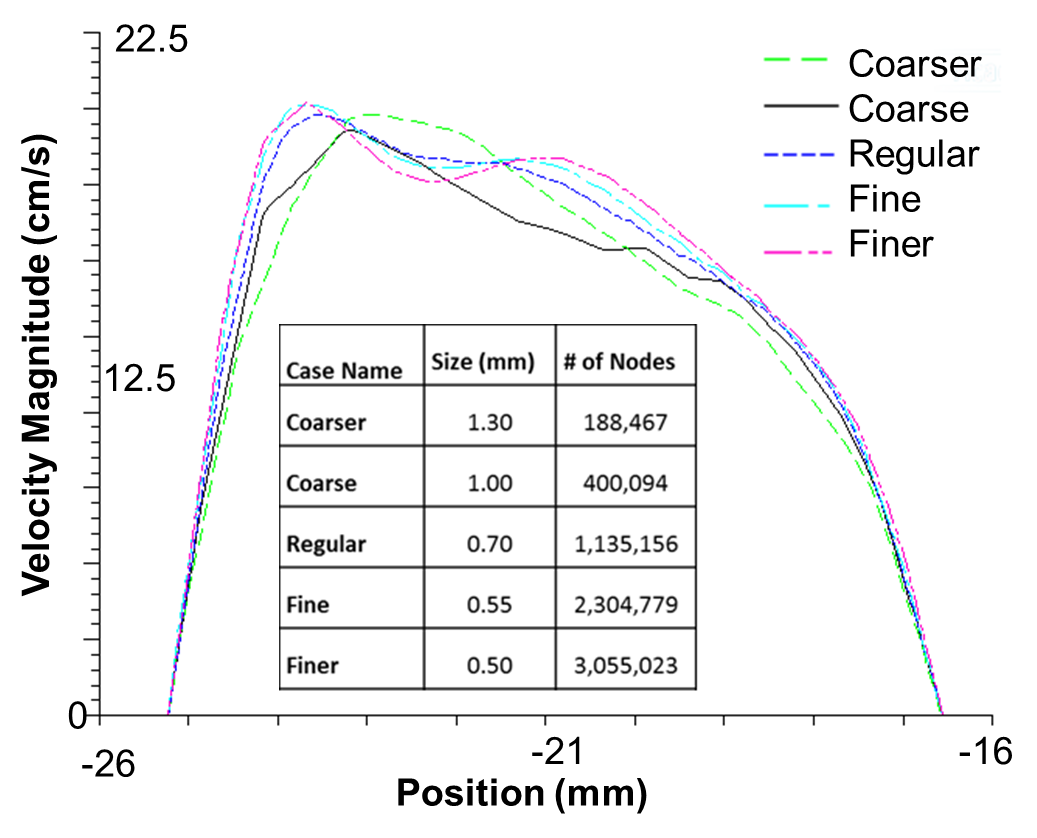
**

**Figure B**: Mesh convergence analysis results in arterial velocity profile. Velocity profiles obtained with different mesh sizes are plotted at the downstream of aortic isthmus (see dashed line in Figure 1 in the Main Text). Five different element sizes and number of nodes are given in table summarizing the mesh convergence analysis. Velocity profiles indicate acceptable convergence and simulations are performed with the regular grid size.
